# A practice improvement package at scale to improve management of birth asphyxia in Rwanda: a before-after mixed methods evaluation

**DOI:** 10.1186/s12884-020-03181-7

**Published:** 2020-10-06

**Authors:** Jacqueline Umunyana, Felix Sayinzoga, Jim Ricca, Rachel Favero, Marcel Manariyo, Assumpta Kayinamura, Edwin Tayebwa, Neena Khadka, Yordanos Molla, Young-Mi Kim

**Affiliations:** 1 Maternal and Child Survival Program, KN 8 Avenue, Rwanda National Police (RNP) Road, Kigali, Rwanda; 2 Rwanda Biomedical Centre, KG 203 St, Kigali, Rwanda; 3 Maternal and Child Survival Program, 1776 Massachusetts Ave, NW, Suite 300, Washington, DC, 20036 USA; 4 Jhpiego Corporation, 1615 Thames St., Baltimore, MD USA

**Keywords:** Newborn resuscitation, Helping babies breathe, Scale up, Quality improvement, Rwanda, Supervision

## Abstract

**Background:**

Helping Babies Breathe (HBB) is a competency-based educational method for an evidence-based protocol to manage birth asphyxia in low resource settings. HBB has been shown to improve health worker skills and neonatal outcomes, but studies have documented problems with skills retention and little evidence of effectiveness at large scale in routine practice. This study examined the effect of complementing provider training with clinical mentorship and quality improvement as outlined in the second edition HBB materials. This “system-oriented” approach was implemented in all public health facilities (*n* = 172) in ten districts in Rwanda from 2015 to 2018.

**Methods:**

A before-after mixed methods study assessed changes in provider skills and neonatal outcomes related to birth asphyxia. Mentee knowledge and skills were assessed with HBB objective structured clinical exam (OSCE) B pre and post training and during mentorship visits up to 1 year afterward. The study team extracted health outcome data across the entirety of intervention districts and conducted interviews to gather perspectives of providers and managers on the approach.

**Results:**

Nearly 40 % (*n =* 772) of health workers in maternity units directly received mentorship. Of the mentees who received two or more visits (*n* = 456), 60 % demonstrated competence (received > 80% score on OSCE B) on the first mentorship visit, and 100% by the sixth. In a subset of 220 health workers followed for an average of 5 months after demonstrating competence, 98% maintained or improved their score. Three of the tracked neonatal health outcomes improved across the ten districts and the fourth just missed statistical significance: neonatal admissions due to asphyxia (37% reduction); fresh stillbirths (27% reduction); neonatal deaths due to asphyxia (13% reduction); and death within 30 min of birth (19% reduction, *p* = 0.06). Health workers expressed satisfaction with the clinical mentorship approach, noting improvements in confidence, patient flow within the maternity, and data use for decision-making.

**Conclusions:**

Framing management of birth asphyxia within a larger quality improvement approach appears to contribute to success at scale. Clinical mentorship emerged as a critical element. The specific effect of individual components of the approach on provider skills and health outcomes requires further investigation.

## Background

Globally, 24% of newborn deaths are due to birth asphyxia [1], which can occur when an infant receives inadequate oxygenation before, during, or immediately following birth. Helping Babies Breathe (HBB) is an evidence-based protocol to manage newborns with birth asphyxia in low resource settings. It is a competency-based educational method that includes immediate essential newborn care and newborn resuscitation skills training and uses hands on practice on anatomical models under direct supervision of a facilitator [2]. HBB has been shown to improve health worker skills and neonatal outcomes at small and moderate scale with intensive support for implementation [3] but there have been problems with retention of skills and sustained improvement in health provider practices [4], especially when implemented under more routine conditions [5].

Recognizing the potential of HBB to improve outcomes related to birth asphyxia, the Rwanda Ministry of Health (MOH) conducted a national scale-up of HBB training in 2011 by integrating it into the Essential Newborn Care (ENC) in-service training module and implementing a stand-alone one-time in-service cascade training of health providers attending births [6]. However, subsequent national level analysis of neonatal death audit information conducted by the MOH in 2012 and 2013 revealed that birth asphyxia remained the leading cause of neonatal mortality [7]. The MOH and other stakeholders identified provider performance and skills retention as the primary reasons for the lack of improvement in birth asphyxia outcomes despite the scale-up of HBB training [7]. A study in Ghana confirmed that although HBB training leads to significant improvement in skills in the short term, retaining these skills over the long term remains a challenge [8].

On-the-job training has been shown to be more effective for acquiring skills and improving performance compared to learning in traditional classroom settings [9]. Clinical mentorship of health facility providers has been shown to improve quality of care and health outcomes, especially when it is part of a multipronged quality improvement strategy and, in fact, this is the focus of the recently published second edition of the HBB strategy [5]. One qualitative study on pediatric quality of care in health centers in rural Rwanda also showed that mentors and mentees found clinical mentorship to be feasible, acceptable and effective at improving quality of care [10]. Studies in South Africa and Botswana also support the effectiveness of clinical mentorship in improving quality of clinical care [11, 12]. Our study aimed to show that a capacity development package focused on mentorship as part of a larger quality improvement strategy would contribute to improved clinical skills and better neonatal outcomes for birth asphyxia at scale.

## Methods

### Setting

At the request of the MOH, the United States Agency for International Development (USAID)-funded Maternal and Child Survival Program (MCSP) co-designed a practice improvement approach and assisted the MOH to implement it in a phased manner across ten of the country’s 30 districts. The MOH chose these ten districts based on their lower than average maternal and newborn health indicators and lack of other partner support. All twelve public hospitals and 160 health centers in the ten districts were included in two phases of program implementation. In the first phase (January – December 2016), four districts received all components of the practice improvement approach while the other six received only the mentorship component. In the second phase (January 2017– June 2018) all ten districts received all components of the approach. In Rwanda, all health facilities provide vaginal delivery and assisted delivery services, with additional sick newborn care and cesarean section services available at the hospital level.

### Overview of the HBB practice improvement approach

**Fig. 1:**
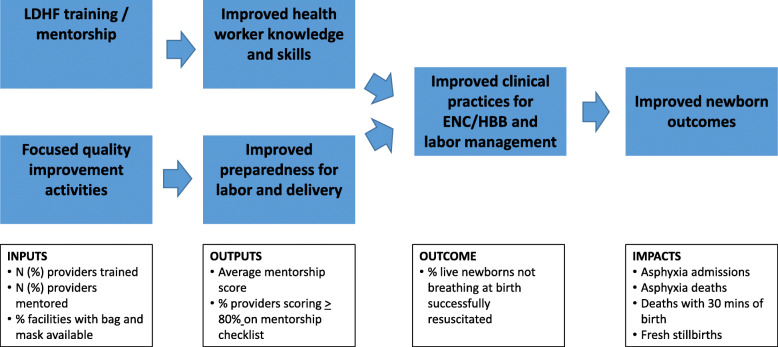
Theory of change for the practice improvement approach

The practice improvement approach involved initial in-service health worker training using a low dose high frequency (LDHF) method which is described elsewhere [13]; ongoing mentorship using NeoNatalie anatomical models for practice using clinical simulations; and quality improvement activities focused on evidence based labor management and preparation for emergencies during and immediately after childbirth. Figure 1 shows the theory of change underlying this practice improvement approach, which is similar to the second edition of the HBB package that was being developed contemporaneously.

### Description of components of LDHF in-service training and mentorship

Seventy-three providers from the 12 district hospitals in the ten districts were selected as potential mentors, because they were already supervisors or they had scored well on an initial assessment of clinical skills for newborn care. They were given an initial three-day off-site orientation to the mentorship activity that included training in mentoring skills and refresher training in HBB. Ensuring the mentors were proficient clinically in training skills consisted of an additional five-day refresher training of comprehensive newborn skills to select the candidates that showed the most promise to be successful mentors. The focus of the 5-day refresher training was to both improve clinical skills and also to improve training/mentoring skills. There was hands- on training at an off-site non-clinical location. Sixty-eight of the participants qualified as mentors in the subsequent post-test.

The selected mentors initially conducted LDHF in-service training with providers in four districts starting in January 2016 (Kamonyi, Musanze, Ngoma, and Rwamagana) and expanded to six additional districts in January 2017 (Gatsibo, Huye, Nyagatare, Nyabihu, Nyamagabe, and Nyaruguru). Due to time and program resources, each public health facility selected two health providers (mentees) who attend births to receive the training. The mentors conducted LDHF in-service training over three sessions of 2 days each, spaced over a 3 week period, with one session per week. For each session, the first day was devoted to theory and the second focused on simulation with anatomical models. The same curriculum used to train mentors was employed. Participants completed pre- and post-tests on knowledge and skills.

**Fig. 2:**
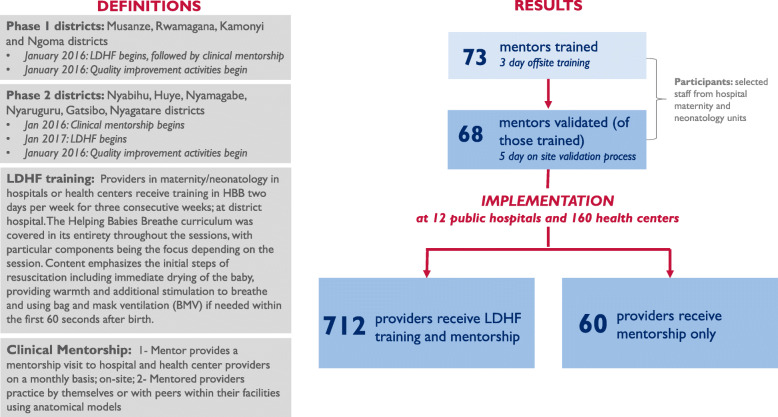
LDHF training and mentorship components in ten districts

District-based mentors visited each health facility at least once a quarter. During each visit, the mentor focused on the two mentees who had or would receive LDHF using an observation checklist to assess progress in skills through observation of simulated resuscitation using a NeoNatalie anatomical model. The mentors also reviewed organization of services and observed other components of facility readiness. In cases where the mentees were not working or could not be located during the day of the mentorship visit, mentors would invite other additional mentees to participate in the mentorship visit. The mentees were also given materials and encouraged to conduct peer-to-peer mentorship activities in between visits from offsite mentors. Sixty-two (8%) of providers received clinical mentorship only without participating in LDHF trainings. Figure 2 shows the rollout of the LDHF training and clinical mentorship components in the ten districts.

### Description of the focused quality improvement component

At the start of activities, all facilities were provided with newborn resuscitation equipment, both for clinical activities and for peer practice and mentorship. As part of LDHF training cascade, mentors and mentees were trained on the tenets of the quality improvement approach and given tools for its implementation. The materials were adapted from pre-existing Ministry of Health tools and aligned for the context of the program intervention. One of the main tools targeted the use of the birth audit asphyxia tools. During clinical mentorship visits, mentors then helped mentees identify gaps in facility and health provider readiness to provide quality newborn services. They discussed the trends in key indicators included in the routine information system with mentees, the facility in-charge, and the quality improvement (QI) committee. Existing QI committees at health centers and hospitals received ongoing coaching from MCSP staff on their regular review of data and formulation of quality improvement action plans. The action plan included quality improvement activities that were formulated to respond to the gaps identified. A report that included both action plans and actions that had been taken since the previous QI committee meeting was then shared with the facility in-charge.

### Description of semi-annual multi-district learning meetings

After 6 months of implementation, with the support of MCSP, the MOH convened semi-annual multi-district learning workshops. Participants included health providers, mentors, and hospital managers from across the districts included in the practice improvement activities, along with representatives from other donor and partner organizations. During this activity, participants shared the challenges they faced and the actions they had taken for practice improvement as well as lessons learned for making mentorship more effective and feasible. The meetings also provided an opportunity for mentors to improve their mentorship skills through hearing about the experiences of their associates.

### Study design and sampling

The study used a mixed methods before-after design to assess changes in selected outputs and outcomes, according to the theory of change. All health providers who received mentorship with MCSP support and all public facilities in the ten implementation districts were included in the assessment.

### Tools, data collection, and data analysis

Baseline (October 2015) and final (May 2018) assessments were conducted for facility readiness to provide newborn services using a tool based on the Service Availability and Readiness Assessment (SARA) [14] and Service Provision Assessment (SPA) [15] and a clinical skills assessment based on standard HBB materials [2].

Input indicators (numbers and percentages of providers trained and mentored) and activity indicators (pre and post training assessments of knowledge and skills) were collected by project staff, the latter using a standardized checklist for essential newborn care and newborn resuscitation. The MOH agreed on a mentorship checklist which was applied during mentorship visits. This checklist was adapted from the pre/post-test training assessment tool and was based on the standard HBB OSCE B materials [2]. All mentors and MOH staff using the checklist underwent training to standardize their clinical observation skills of the competencies included in the assessment. Project staff worked with MOH counterparts to perform spot checks on use of the checklist and maintain its standardized use across districts and facilities. Providers were counted as competent once they achieved a score of 80% or above.

Data on clinical practices and outcomes relevant to the theory of change were obtained from service statistics routinely reported in the Rwanda health management information system on a monthly basis. The indicators analyzed were the percent of newborns not breathing at birth who were successfully resuscitated, the number of asphyxia admissions to Neonatal Intensive Care for asphyxia, deaths due to asphyxia, deaths within 30 min of birth, and fresh stillbirths. These variables were included because they are outcomes that the study team expected to see improve with the implementation of a practice improvement intervention, as depicted in the theory of change (Fig. 1). The indicators were analyzed quarterly in Excel by project staff and facility data managers. District level MOH staff conducted monthly data reviews as well as quality assessments on a quarterly basis with support from MCSP. The results were also reviewed by members of the MOH-led national Newborn Sub-Committee. For the summative analyses of all outcome variables for this paper, time-value plots were made, slopes calculated, and linear regressions were run to test for the significance of the slope. A chi square test for trend was employed for analysis of the evolution of the knowledge and skills test with health providers.

Key informant in-depth interviews with facility managers, mentors and mentees on the practice improvement approach were conducted at the end of the practice improvement activities at the end of the project. Transcripts of the interviews were imported into Atlas.ti for analysis. A list of codes was developed in advance of the interviews, and during analysis additional codes were developed. Participant quotes and general responses were linked to codes to summarize the main themes.

### Ethical considerations

The assessment protocol was reviewed and given a waiver by the Rwanda National Ethics Committee. The protocol was also determined to be non-human subjects research by Johns Hopkins University School of Public Health Institutional Review Board, as it was deemed a quality improvement activity. Ethical guidelines applicable to Human Subjects Research were nonetheless followed during data collection. Written informed consent was obtained from all qualitative participants.

## Results

Table 1 shows the profile of the health facilities in the ten districts implementing the practice improvement approach before activities began. Data on the annual number of deliveries were obtained from the HMIS.

**Table 1:** Baseline (2015) profile of health facilities in ten districts implementing the practice improvement approach

District	Number of health centers	Number of hospitals	Number of deliveries in 2015
**Phase 1 districts**			
Rwamagana	14	1	9187
Ngoma	12	1	9478
Kamonyi	13	1	7029
Musanze	15	1	12,410
**Phase 2 districts**			
Huye	16	1	10,239
Nyamagabe	19	2	8805
Nyaruguru	16	1	6247
Gatsibo	19	2	14,825
Nyagatare	20	1	14,941
Nyabihu	16	1	8405
**Total**	**160**	**12**	**101,566**

Source: Rwanda HMIS

### Input level variables: service readiness

At baseline in 2015, 35% of health centers and 67% of hospitals had newborn resuscitation equipment (bag and mask) and at endline in 2018, 98% of health centers and 100% of hospitals had at least one bag and mask (newborn size) at the facility. During implementation of the practice improvement approach 993 (51%) of the estimated 1960 eligible^1^ providers in the maternity and neonatology units in the 172 health facilities were recruited for clinical mentorship and given an initial assessment in HBB, 95% (943/993) of whom were nurses and 5% (50/993) of whom were midwives. Over three quarters (78%, or 772/993) began the mentorship process, 712 (92%) of whom also received LDHF.

### Output level variables: improvements in provider knowledge and skills

The average pre-test skills and knowledge score, conducted before the initial LDHF training session for providers, was 44% and the average post-test score was 88%. The clinical knowledge and skills assessment applied at the end of the project included 64 providers randomly selected from the 172 health facilities in the initiative. These providers scored an average of 85% on HBB skills and knowledge using the same assessment tool used during mentorship. It should be noted that the providers for the endline assessment were selected among *all* providers, whether they had directly received offsite mentorship or not, as all providers at least received peer-to-peer mentorship.

**Fig. 3:**
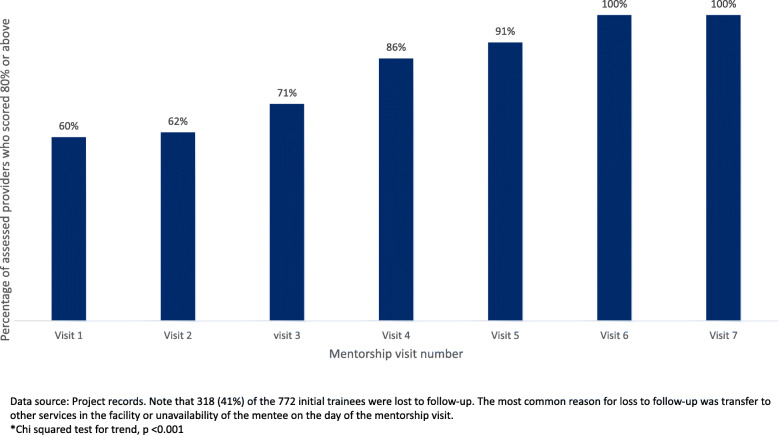
Percentage of assessed providers who scored 80% or above* on HBB skills and knowledge

Figure 3 shows the percentage of assessed providers who scored 80% or above at each mentorship visit. The majority (60%) of mentees passed (i.e., received a score of 80% or above) on the first mentorship visit; 86% by the fourth visit; and 100% by the sixth visit. In other words, after one mentorship visit, 60% of mentees were competent. An average of 9% more mentees passed with each mentorship visit until 100% of assessed mentees had. After obtaining a passing score, a subset of 220 learners were followed for an average of 5 months. Nearly all (98%) of the participants maintained or improved their skills.

### Outcome variable: improvement in clinical practice

**Fig. 4:**
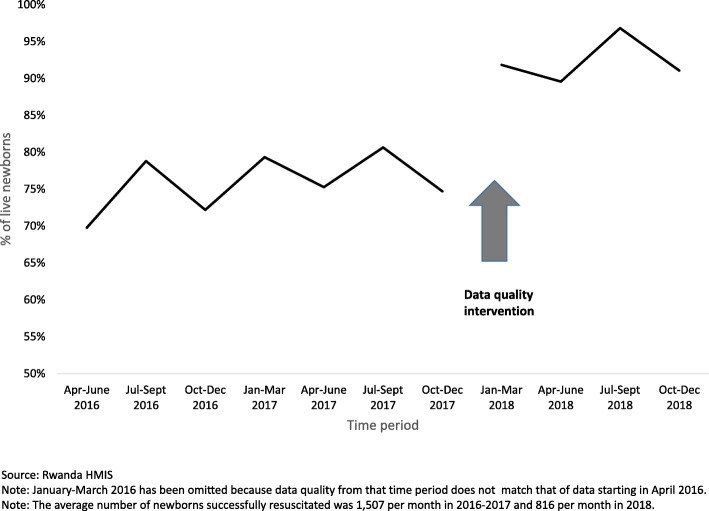
Percentage of live newborns not breathing successfully resuscitated in 10 implementation districts

Figure 4 shows the percentage of live newborns not breathing at birth successfully resuscitated. The percentage increased from 70% in April–June of 2016 to 91% in Oct- Dec of 2018. There is a notable rise in the success of resuscitations reported from the last quarter of 2017 to the first quarter of 2018. This coincided with an intensive data quality intervention. After supervisors had repeatedly noted that there was inconsistency among providers in whether they reported tactile stimulation as the first step in resuscitation, a decision was made that providers would only report those resuscitations in which they used bag and mask. This guidance did not change the definition of the indicator in HMIS, but rather clarified it, and was disseminated to all districts and providers across the country. This emphasis on correct reporting resulted in a nearly 50% drop in the number of resuscitations reported, and a 17% rise on the proportion of successful resuscitations.

### Improvements in health outcomes

Three of the four relevant facility level newborn health outcomes tracked in the HMIS improved between 2015 and 2018, and the improvement in the fourth indicator just missed statistical significance (Table 2). Neonatal admissions due to asphyxia showed the largest improvement, from 22.0 neonatal admissions due to asphyxia per 1000 live births in 2015 to 13.9 in 2018. This was an annual reduction of 12.9% (95%CI = 8.3–17.5%). Neonatal deaths due to asphyxia at health facilities decreased from 3.8 deaths per 1000 live births to 3.3 deaths per 1000 live births, representing an annual reduction of 4.2% (95%CI = 0.2–6.3%). The number of fresh stillbirths per 1000 deliveries decreased from 10.2 in 2015 to 7.5 in 2018, a 6.2% annual reduction (95%CI = 2.1–11.4%). Death within 30 min of birth, although not a global standard, is routinely reported in the HMIS. It is meant to capture deaths that are most likely due to asphyxia. Death within 30 min of birth decreased from 3.2 deaths per 1000 live births to 2.6 deaths per 1000 live births, a 4.0% annual reduction. This just missed statistical significance, with the 95%CI crossing zero (95%CI = + 1.2% to − 8.0%), which was not statistically significantly different from no decrease (*p* = 0.06).

**Table 2:** Newborn health outcomes at health facilities in the ten implementation districts, 2015–2018

	Year				Annual improvement, absolute and percent	95% Confidence Interval
	2015	2016	2017	2018		
Neonatal admissions to the Neonatal Intensive Care Unit due to asphyxia per 1000 live births	22.0	20.9	13.4	13.9	−2.8 (12.9%)	−1.8 to −3.8 (8.3–17.5%)
Neonatal deaths at the health facility due to asphyxia per 1000 live births	3.8	3.2	2.9	3.3	−0.2 (4.2%)	− 0.01 to − 0.3 (0.2–6.3%)
Fresh stillbirth at the health facility per 1000 deliveries	10.2	9.7	9.4	7.5	− 0.6 (6.2%)	− 0.2 to − 1.1 (2.1–11.4%)
Newborn Deaths within 30 min of birth at health facilities per 1000 deliveries	3.2	2.8	3.0	2.6	−0.1 (4.0%)	+ 0.03 to 0.2* (+ 1.2% to − 8.0%)

*p = 0.06

### Provider perspectives on mentorship

Interviewed providers expressed satisfaction with the mentorship approach, stating that they felt more confident to provide care to clients after receiving support from mentors. Respondents noted that patient flow and logistics within the maternity ward also improved, contributing to effective triage and management of emergency cases. One midwife noted that after her hospital received training and mentorship, critical cases in the maternity ward were managed in a designated space with dedicated staff.

*‘Before we had no place reserved for maternal emergencies. We did not give much considerations to those emergencies. They shared the same wards with other cases. After training and mentorship, we created a room for critical cases, and we allocated staff to take care of them. The triage is done and the critical cases are placed in that room for special care.’ – Hospital midwife*

Clinical staff felt that newborn resuscitation skills improved after mentoring was implemented. One provider noted that newborns in distress frequently died because clinicians did not correctly apply the resuscitation approach. With the ability to practice and receive real-time advice from a mentor, health providers felt that newborn resuscitation was being employed correctly and was saving the lives of newborns who might not have previously survived.

*‘Before, a baby with fetal distress died easily because we lacked knowledge about how to help the baby to breathe or how to do resuscitation. We were all ignorant in the matter of Apgar scores, and resuscitation was done without following resuscitation steps, but today, no child dies due to birth asphyxia. We try our best; we resuscitate; we call for help; and we save lives.’ - Health Center Midwife*

Data use for decision making is another area that providers felt had improved after they received support and guidance. Relevant service statistics and quality of care measures were not routinely collected before the intervention was implemented. One staff member noted that her colleagues appreciated the value of quality data and the key role it can play in decision making at the facility level.

*‘Before it was very hard to get actual data. Staff didn’t know why they needed to collect all these data. You would ask for the number of deliveries done and receive data which was not related to reality, simply because they didn’t know the importance of reporting good data. After receiving capacity building and data management … changes are remarkable, everyone knows how to collect good data and its importance in decision making.’ - Hospital Maternity Matron*

*‘Before submitting a certain report, I check and ensure the quality of my data. One time we found that the number of partograms was not equal to the number of normal deliveries. There were missing partograms, we discovered that the missing partograms were attached to transfer notes. We made copies of the missing partograms and showed the issue to the staff.’ - Health Center Midwife*

*‘I have been a titulaire in different health centers for more than 16 years, we’ve had several trainings in various domains, topics or subject but once you left the training facility it was finished, no one asked you what you studied so that you share it. And most times you already even forgot about it, but with the LDHF approach, there is a difference; the training was happening onsite, more staff benefited in terms of theory and practice as it was done on site. Cases would be managed even during the training.’*

*- Health Center Titulaire*

## Discussion

We did not compare our results to non-intervention districts because several MOH and implementing partner interventions were taking place in non-intervention districts, making them imperfect controls. In addition, the ten intervention districts received more support for data quality improvement compared to the other 20 non-supported districts. In fact, improving data quality was a focus of the mentorship visits and the multi-district learning meetings in the project supported districts, which were activities that did not take place in the 20 non-supported districts. Even with these interventions to improve data quality, there were persistent issues related to which newborns should be recorded as *in need of resuscitation*. This continued to affect the quality of the routine data, especially for the percent of newborns successfully resuscitated. As seen in Fig. 4, the proportion of successfully resuscitated newborns remained fairly flat during the first seven quarters, followed by a jump from 75 to 92% between the last quarter of 2017 and the first quarter of 2018. This increase corresponds with provider mentoring that emphasized the definition of the data elements for the newborn resuscitation indicator should only include cases where a bag and mask was used. Mentors also reminded mentees that stillbirths should not be included in either the numerator or the denominator. Revisions of the HMIS reporting form and the printing of registers allowed for a single reporting tool that included the updated newborn resuscitation indicators and enabled ease of reporting. Although providers received this guidance in Rwanda’s other 20 districts, they did not receive the project supported mentorship focused on newborn outcomes that emphasized adherence to the guidance.

Three of the four health outcomes of interest improved, and the improvement in the fourth just missed statistical significance. Lending credence that the improvements in outcomes can be attributed to the quality improvement approach is the fact that the level of newborn deaths due to asphyxia was increasing in the years before the intervention (2013–2014) in the intervention districts. Moreover, nationwide in 2013, 39% of neonatal deaths were due to asphyxia, making it the leading cause of newborn death in Rwanda, whereas by 2018 the proportion of newborn deaths due to asphyxia decreased to 26% nationwide [16].

Few other studies evaluating HBB have been done at as large a scale (33% of the population of Rwanda) and under as routine conditions [17], but we are aware of a recently published stepped wedge study of HBB embedded in a similar QI package in 12 hospitals in Nepal showed a reduction in intrapartum-related mortality [18]. A previous nationwide study of implementation in Tanzania, documented retention of skills but did not look at clinical practices or newborn outcomes. The intervention there also consisted of on-the-job training and supportive supervisory visits which were associated with improvements in skill retention [19].

As posited by the recently published second edition of HBB materials, framing HBB in a larger quality improvement context appears to be critical to its success at scale. The previous scale up of HBB in 2011 in Rwanda was mainly focused on health provider training, but newborn deaths due to birth asphyxia remained high afterwards until initiatives started which included improvements in basic equipment, data use, and management/government mechanisms.

Although the assessment was not designed to show the separate effects of each of the components of the practice improvement approach, there was some evidence that clinical mentorship was the critical component. There was no significant difference across districts related to the sequencing of mentorship with other components of the approach, and unexpectedly we found that the improvements in outcome indicators in the six Phase 2 districts started immediately in 2016 even when they had only received the mentorship component. Further, though 456 of the estimated 1960 providers in the maternity units received two or more mentorship visits, improvements in provider practice and newborn outcomes were seen across all maternity providers in the facilities. Although the mentors experienced difficulties because those originally trained were not always available for ongoing mentorship, their occasional absence in the facility allowed them to mentor other providers. While this happened as an adaptation of the initially planned rollout approach, it actually had the effect of benefitting a larger number of providers with mentorship and practice sessions. Peer to peer mentorship implemented between on-site mentorship visits may be another important factor in these successes. The improving percentage of mentees who scored 80% after each mentoring visit and the improvement or maintenance of skills among providers previously deemed competent also lends credence to the importance of mentoring for retention of skills among the entire group of health workers at a facility. In the interviews, facility staff identified mentorship as the component that they felt most helped them implement the practice improvements, including strengthening the functioning of their team, strengthening referral networks from health centers to hospitals, and motivating them to feel that achieving practice improvement was possible. This finding is consistent with the World Health Organization’s Quality of Care Framework guidance, which states that a positive attitude of a health worker can influence appropriate use of effective clinical and non-clinical interventions [20].

Assuring the availability of the necessary equipment to perform resuscitation is critical to the success of improved provider skills and newborn outcomes. This was noted by MOH staff as an important element of continued advances in quality of care at the facility level.

Activities that improved continuous learning above the level of the individual provider also appeared to be crucial. Mentorship efforts included review of data and quality improvement plans included efforts to improve data quality. This created a positive feedback loop, whereby the established facility quality committees and district managers began to trust and therefore use their data more. This was clear in the in-depth interviews with providers and managers. We feel that the semi-annual multi-district learning meetings were also critical, as it reinforced the mentorship and learning at the facility level. Mentors and their leaders noted that they thought the experience sharing at these meetings helped improve their practice and increase their motivation.

### Limitations

This assessment had a number of limitations. First and foremost, as already stated, we were not able to compare results in the ten intervention districts to the other 20 non-intervened districts because of the differential intensity of data quality improvement efforts. Also, key informants may have been reluctant to give negative information about the program and its interventions. The project worked to overcome this issue by using external interviewers. While it took providers a variable number of mentorship visits to be deemed competent, the project was not able to investigate the underlying reasons for these variations. Future research could analyze these reasons to inform more effective scale up of the intervention. Finally, although the study team reviewed contextual information, the effects of other factors within the health system on observed improvements in provider skill and newborn outcomes could not be systematically controlled for.

## Conclusions

This assessment gives plausible evidence that a system-oriented practice improvement approach was associated with improved health worker knowledge and skills; improved clinical practices; and improvements in newborn health outcomes. However, the study cannot demonstrate the separate effects of each of the components of the approach. This gives one of the first demonstrations of how HBB might function effectively at scale under relatively routine conditions. In order for the approach to be institutionalized, the central and district levels will need to include the modest incremental costs of mentorship in their annual plans, especially to promote regular peer-to-peer mentorship.

## Supplementary information

**Additional file 1:** **Supplementary Material 1.** Final mentorship data level 1. Mentorship scores of mentees over the course of mentorship visits, throughout project implementation.

**Additional file 2: Supplementary Material 2.** Final level 2 and 3 data. Changes over time in the % of successfully resuscitated newborns. Changes over time in newborn health outcomes.

## Data Availability

The baseline facility readiness assessment has been uploaded to the USAID Data Depository (DDL) and is publicly available at https://data.usaid.gov/Maternal-and-Child-Health/2015-Health-Facility-Assessment-in-Rwanda-for-Mate/2wzp-snem. All data generated during this study are included in this published article [and its supplementary information files].

## Abbreviations

ENC: Essential Newborn Care
HBB: Helping Babies Breathe
HMIS: Health Management Information System
LDHF: Low Dose High Frequency
MCSP: Maternal and Child Survival Program
MOH: Ministry of Health
OSCE: Objective Structured Clinical Examination

## Footnotes

1 Eligible providers include all clinical staff in the maternity and neonatology units in the 172 MCSP supported facilities. This includes an estimated 1960 providers.
